# Supplementary material for: Effect of Radiotherapy in Addition to Surgery in Early Stage Endometrial Cancer: A Population-Based Study
Source: Cancers (Basel). 2020 Dec 17;12(12):3814. doi: 10.3390/cancers12123814 (PMC7766752; doi:10.3390/cancers12123814)
Supplement: Supplementary file 1 [file cancers-12-03814-s001.pdf]

cancers

MDPI

Supplementary Materials:

Effect of Radiotherapy in Addition to Surgery in Early Stage Endometrial Cancer: A Population-Based Study

Daniel Medenwald, Susan Langer, Cornelia Gottschick and Dirk Vordermark

Table S1. Sensitivity analysis considering all early stage T1, N0/x, M0/x cases. The model is adjusted for age, stage (T1a, T1b and T1), grading, and chemotherapy (CT).

Parameter	Sensitivity Analysis	Sensitivity Analysis
RT-	Ref.	Ref.
RT+	0.84 (0.77–0.92)	0.93 (0.83–1.05)
Age (10 years)	1.07 (1.07–1.08)	1.07 (1.07–1.08)
Stage IA *	Ref.	Ref.
Stage IB **	1.36 (1.25–1.49)	1.51 (1.35–1.7)
Stage I	1.32 (1.01–1.74)	1.32 (0.93–1.88)
Grade 1	Ref.	Ref.
Grade 2	1.23 (1.11–1.35)	1.21 (1.1–1.33)
Grade 3	2.18 (1.95–2.44)	2.15 (1.92–2.41)
CT-	Ref.	Ref.
CT+	1.81 (1.39–2.36)	1.82 (1.39–2.37)
Stage/RT Interaction †		0.8 (0.67–0.94)
Stage/RT Interaction ††		1 (0.57–1.75)

* Stage IA (TNM version 8); ** Stage IB (TNM version 8), † IA and no RT as the reference for the interaction. Interaction estimate refers to the effect of RT in Stage IB. †† Interaction estimate refers to the effect of RT in Stage IA, G1/G2.

Table S2. Sensitivity analysis of all case with endometrioid carcinomas considering all early stage T1, N0/x, M0/x cases. The model is adjusted for age, stage (T1a, T1b and T1), grading, and chemotherapy (CT).

Parameter	Hazard Ratio
RT-	Ref.
RT+	0.94 (0.82–1.08)
Age (10 years)	1.08 (1.07–1.08)
Stage IA *	Ref.
Stage IB **	1.56 (1.37–1.78)
Grade 1	Ref.
Grade 2	1.17 (1.05–1.3)
Grade 3	2 (1.74–2.3)
CT-	Ref.

CT+	2.28 (1.64–3.18)
Stage/RT Interaction †	0.75 (0.61–0.92)

* Stage T1a (version 8); ** Stage T1b (version 8), † T1a and no RT as the reference for the interaction. Interaction estimate refers to the effect of RT in Stage T1b (T1c).

Table S3. Sensitivity analysis differentiating between cases with histopathological examination (pN) vs. no histopathological examination of lymph nodes (cN). The model is adjusted for age, stage (T1a, T1b and T1), grading, and chemotherapy (CT).

Parameter	Histopathologic Examination (HR)	No histopathologic Examination (HR)
RT-	Ref.	Ref.
RT+	0.98 (0.83–1.16)	0.89 (0.75–1.05)
Age (10 years)	1.07 (1.07–1.08)	1.07 (1.07–1.08)
Stage T1a *	Ref.	Ref.
Stage T1b **	1.69 (1.44–1.98)	1.34 (1.13–1.6)
Grade 1	Ref.	Ref.
Grade 2	1.32 (1.16–1.5)	1.12 (0.96–1.3)
Grade 3	2.11 (1.77–2.51)	2.19 (1.86–2.58)
CT-	Ref.	Ref.
CT+	1.86 (1.19–2.91)	1.8 (1.28–2.52)
Stage/RT Interaction †	0.76 (0.59–0.97)	0.88 (0.69–1.12)

* Stage T1a (version 8); ** Stage T1b (version 8), † T1a and no RT as the reference for the interaction. Interaction estimate refers to the effect of RT in Stage T1b.
